# Pacific Salmon and the Coalescent Effective Population Size

**DOI:** 10.1371/journal.pone.0013019

**Published:** 2010-09-27

**Authors:** Can Cenik, John Wakeley

**Affiliations:** Department of Organismic and Evolutionary Biology, Harvard University, Cambridge, Massachusetts, United States of America; University of Umeå, Sweden

## Abstract

Pacific salmon include several species that are both commercially important and endangered. Understanding the causes of loss in genetic variation is essential for designing better conservation strategies. Here we use a coalescent approach to analyze a model of the complex life history of salmon, and derive the coalescent effective population (CES). With the aid of Kronecker products and a convergence theorem for Markov chains with two time scales, we derive a simple formula for the CES and thereby establish its existence. Our results may be used to address important questions regarding salmon biology, in particular about the loss of genetic variation. To illustrate the utility of our approach, we consider the effects of fluctuations in population size over time. Our analysis enables the application of several tools of coalescent theory to the case of salmon.

## Introduction

Pacific salmon include several commercially important, and endangered species. These semelparous species exhibit complex life histories. They typically spend several years in the ocean, then come back to fresh water to reproduce and die [1]. Reproduction typically happens once a year and spawning generally takes place in late summer to early fall [2]. Adults of Pacific salmon invariably die after reproduction but individuals of different ages might reproduce in the same breeding season. This life history poses challenges for the application of standard population genetics tools since the population dynamics are not captured by either the standard overlapping generation model [3], [4] or the standard non-overlapping generation model [5], [6]. In particular, it is of interest to understand how the ancestral genetic process called the coalescent [7], [8], [9], [10] applies to salmon.

Salmon population sizes are diminishing as a result of fishing and loss of habitat. Recently, the role of hatcheries in restoring endangered salmon populations to sustainable levels has been debated [11]. Conservation efforts are guided by both demography and genetics. A successful conservation strategy should take the ecology of the species into account and evaluate the minimum population size required for persistence for a specific amount of time with high probability [12]. Yet, management of decreasing genetic variation is also critical for conservation efforts, and population genetic models have played an important role in developing better conservation strategies [13], [14]. We analyze such a genetic model using the theory of the coalescent.

Coalescent theory is a sample-based approach and is concerned with making inferences about the genetics of populations under a statistical framework [15]. The coalescent is a backward-time stochastic process that models the ancestry of a sample of size *n*, back to the most recent common ancestor of the entire sample [7], [8], [9], [10]. The coalescent makes detailed predictions about patterns of genetic variation in the sample, and it has been invaluable for understanding the effects of genetic drift and other evolutionary forces in a broad range of models, including the Wright-Fisher model and the Moran model [7], [8]

In his seminal papers, Kingman [7], [8] analyzed the process of joining of lineages backward in time and showed that the genealogy of a sample is described by a simple stochastic process that he called the *n*-coalescent. The coalescent is a continuous-time Markov process that exists in the limit as the population size *N* tends to infinity with time rescaled in a particular way [8]. In the case of the Wright-Fisher model, time is measured in units of *N_e_* = *N* generations [8]. In other models *N_e_* is proportional to *N* [16]. When the coalescent exists in the limit 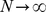, with time rescaled by *N_e_*, the ancestry of a sample of finite size (*n*) is composed of *n*−1 independent, exponentially-distributed coalescence times, each ending with a coalescent event between a random pair of lineages. When there are *i* ancestral lineages the rate of coalescence is *i*(*i*−1)/2 and the expected time to a coalescent event is 2/(*i*(*i*−1)). For a review of coalescent theory, see [17] or [18].

One of the advantages of Kingman's coalescent is its robustness [19]. It is the limiting ancestral process for a diverse array of population models [20], [21]. A theory about Markov processes with two time scales by Möhle is very useful in obtaining such results [21], [22]. The coalescent has been established for models that involve diploidy and two sexes [22], strong migration [23], partial selfing [21], [24], and for populations composed of very many subpopulations [25]. These studies have concluded that if time is appropriately scaled then the distribution of the time to common ancestry is the same as that of Kingman's standard coalescent model.

The concept of rescaling of time in the coalescent process naturally leads to a new type of effective population size. A population is said to have a coalescent effective population size (CES) if its ancestral process converges to the Kingman coalescent in the limit as the population size tends to infinity with time measured in units of *N_e_* generations [16], [20].

In general, the effective size of a population is defined as the size of an ideal (Wright-Fisher) population that would show the same behavior as the population of interest, in terms of loss of genetic variation due to random drift [6], [26]. Depending on the measure of genetic drift considered, three different effective population sizes have previously been defined: variance effective size [27], eigenvalue effective size [28], [29] and inbreeding effective size [6], [26]. Sjödin *et al.* (2005) argued that the CES should be preferred over other definitions of effective size, because its existence implies that all aspects of genetic variation in samples of any size should be consistent with the predictions of Kingman's coalescent.

In this paper, we study a model of a typical Pacific salmon life history. We assume that there are two juvenile and three adult age classes, but the results generalize readily to other numbers of age classes. We analyze the backward-time ancestral process, and prove convergence to a continuous-time coalescent process. The proof is based on a theorem for Markov chains with two time scales [21], [22] which establishes weak convergence to the standard Kingman's coalescent with the appropriate rescaling of time. We deduce the coalescent effective population size and discuss the significance of this quantity in understanding the biology of salmon. We also extend the theory to include changes in population size over time, and obtain results that are helpful in interpreting some previously reported computer simulations [30].

## Methods

### 1. Model

We will use a generalized Wright-Fisher model that aims to capture the life cycle of Pacific salmon, and is similar to previous models for semelparous organisms [30], [31], [32]. We consider an age-structured, haploid population of size *N*. In most population genetic models, the population size refers to the sum of all individuals at a particular time. To illustrate our definition of population size *N*, let us consider an example. In each reproductive season, thousands of eggs are laid yet only a tiny fraction of these eggs survive beyond the early stages of development. In a chinook salmon population, an average female lays 4000 eggs and age-specific survival values, s(x), are 0.05, 0.1, 0.8, 0.8, 0.8 (following the parameters estimated in [33]). Now, consider that 500 females laid eggs in a given breeding season. Out of 200,000 eggs that were laid, 10,000 offspring will hatch to be in the first age class. Of these 10,000 offspring, 1000 will survive to the second age class. Given that 80 percent of these will survive to ages 3, 4, and 5, there would be 800, 640 and 512 in these age classes, respectively. Therefore, the total population size *N* would be equal to 10,000+1,000+800+640+512 = 12,952. Thus, we disregard those 190,000 individuals that did not make it into the first age class. The theory that we develop below is not closely tied to how the population size is defined, though. Any consistent definitions of the population size could be adopted as all parameters of the model are defined relative to the total population size *N*.

**Figure 1:**
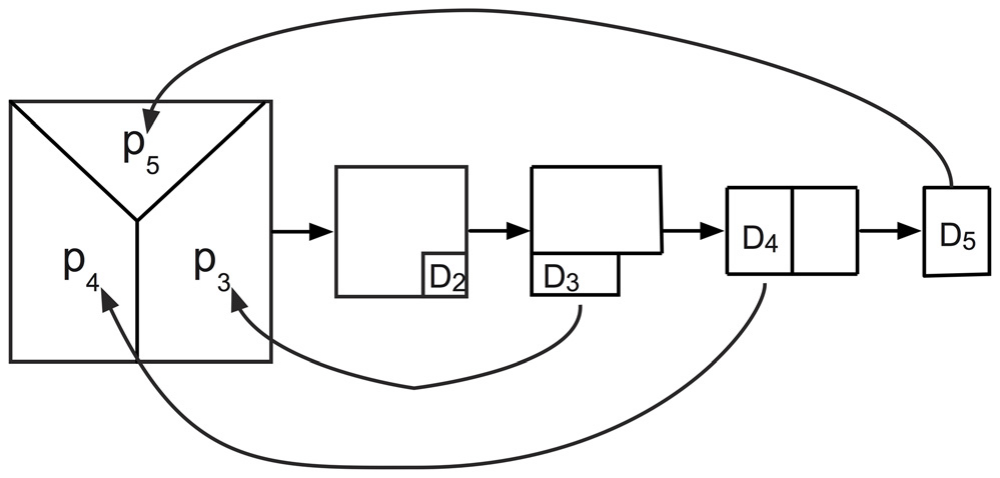
Schematic drawing of the model. The arrows represent the evolution of the system in one time step forward in time. The individuals labeled with *D_i_* represent the group that does not survive to the next age class. The *D_1_* individuals that do not survive to the second age are not shown but implied in the figure. The proportion of age class one produced by these individuals are represented with *p_i_*.

For concreteness, the population is divided into five age classes. We assume that no reproductively active individual survives beyond age five even though a more general treatment with an arbitrary number of age classes is possible. The number of individuals in age class *i* is denoted by *N_i_*. For spring chinook salmon, empirical data from Marsh Creek in Central Idaho suggests that the spawners are between ages three and five [34] so we assume that individuals in the age classes one and two are juveniles. These juveniles generally spend close to two years in freshwater before moving to the ocean. Hence, all the newborns have parents that are either three, four or five years old and we denote the proportion of newborns that have been produced by parents in age class *i* by *p_i_* such that 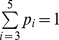 (Figure 1).

A critical assumption of this model is that the total population size *N* is constant over time. This is an unrealistic assumption in general and especially for salmon where fluctuations in the population size are known to be a major factor in reducing genetic variation [30]. Below, we relax this assumption to account for rapid fluctuations in the population size. Constant population size assumption also implies that the number of individuals in each age class and the proportion of the newborns that are parented by individuals in the different age classes remain unchanged. In the next section where we allow fluctuations in population size over time, we similarly assume the number of individuals in each age class to be linearly proportional to the total population *N* at time *t*.

Each year a certain proportion of the population dies either after reproducing or due to other causes. We denote the number of individuals that die in a given age class *i* by *D_i_*. Since the total population size and the age structure is constant through time, the size of age class *i+1* is equal to *N_i_*_+1_ = *N_i_*−*D_i_* for all *i* less than five. Furthermore, no individual in the population survives beyond age five and hence *N*_5_ = *D*_5_. For convenience, we introduce the parameter *c_i_*, which is defined as the number of individuals dying in age class *i* that die each year divided by the total population size *N*. In the limit as the total population size tends to infinite, the following relation holds
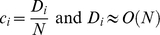
where *O(N)* represents the standard order notation.

Since none of the individuals spawning in generation *t* are able to survive to generation *t*+1, the parents of all newborns are among the *D_i_* dying individuals. Within each age class, reproduction is according to the Wright-Fisher model such that any one individual out of the *D_i_* adults is equally likely to be the parent of each of the *p_i_N*_1_ offspring produced by the adults in the *i* ^th^ age class. In the Wright-Fisher model, the number of offspring of an individual follows a multinomial distribution. Therefore, the expected number of offspring produced by adults in a given age class is equal to *p_i_N*_1_/*N_i_*. Similar models were previously used to analyze the population genetics of semelparous organisms using forward-time approaches [30], [31], [32], [35], [36], [37], [38], [39], [40].

To understand how the *c*_i_ parameters are calculated, let us consider the same example used to illustrate the calculation of population size *N*. In that example, the age-specific survival values, s(x), were 0.05, 0.1, 0.8, 0.8, 0.8. The total population size *N* was 12,952 and there were 10,000, 1,000, 800, 640, and 512 individuals in age classes one through five, respectively. In this example, *c*_i_ parameters would be 0.69 (9,000/12,952), 0.02 (200/ 12,952), 0.01 (160/12,952), 0.01 (128 / 12,952), and 0.04 (512/ 12,952). In more general terms, the age-specific survival values s(x) are related to *c*_i_ parameters as follows:
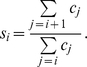



#### (i) The Ancestral Process

We will consider a sample of two lineages from age classes *i* and *j*, where *i* and *j* are not necessarily distinct and we follow the distribution of the time to their common ancestor in this population. First, let us consider a just single lineage that is not in age class one. In the previous year, this lineage must have been in age class *i*−1. When the lineage is in age class one in the current year, it must be a direct descendent of a lineage in age class *k* with probability *p_k_* (See Table 1 for a list of definitions of parameters). This single-lineage process defines a discrete time Markov chain with the following transition matrix:
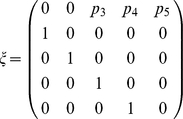
Note that all states of this Markov chain can be visited after a finite number of time steps. Also, each state has a positive probability of occurrence after a finite number of transitions. These properties imply that this is an irreducible, recurrent and aperiodic Markov chain.

**Table 1:** Definition of the terms used in the paper.

Parameters	Definition
*N*	Total population size
*N*_e_	Effective population size
*N*_b_	Effective number of breeders
*N*_i_	Number of individuals in age class *i*
*c*_i_	Ratio of individuals dying at age *i* to the total population size
*D*_i_	Number of individuals dying at age *i*
*p*_i_	The proportion of newborns produced by parents in age class *i*
*ξ*	Transition matrix of the Markov chain that describes the movement of a lineage among age classes backwards in time
⊗	Kronecker product
***M*** *_N_*(*t*)	Population size at year *t*
*ϕ*	Proportion of individuals in the first age class to the total population size
*g*	Generation Length
***X*** *_i_*	Proportional contribution of spawners in year *i* to the next generation

We now consider the ancestral process of two lineages sampled randomly without replacement from the population. An added complication, and the focus of our interest in this case, is the possibility that these two lineages coalesce. At any generation *t*, each of these two lineages can be in any of the five age classes or they could have coalesced (in which case one need not keep track of which age class they are in). This gives a total of 26 possible states. We order these 26 possible states lexicographically such that the sample space (Ω) will be:
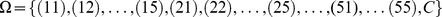
State (*ij*) means that the first lineage is in age class *i* and the second one is in *j* and they have not already coalesced. Note that we have distinguished the state in which the first lineage is in age class *i* and the second lineage is in age class *j* from the state in which the first lineage is in *j* and the second is in *i*. The state *C* indicates that the two lineages have reached their common ancestor and coalesced.

Once the lineages coalesce they remain in that state indefinitely and therefore the coalescent state *C* is an absorbing state of this stochastic process. Also note that given enough time the lineages can move to any of the other 25 states if they have not already coalesced. Due to the particular nature of the semelparous life style, a pair of lineages can coalesce at time step *t* if and only if they are both in the first age class in time step *t−1*.

#### (ii) Weak Convergence to Kingman's Coalescent

In this section, we prove that the ancestral process for a sample size of two converges to a continuous time process with rate of coalescence equal to one, as in Kingman's coalescent. We obtain the time scale, or effective population size, of this process using a convergence theorem for Markov chains with two time scales by Möhle (1998a, 1998b). Möhle considered a sequence of discrete time homogeneous Markov chains 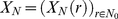 with finite state space and transition matrix:
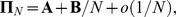
where the transition matrix is decomposed into two matrices ***A*** and ***B*** such that:
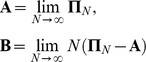



He showed that if ***A***, ***B***, and 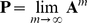 exist then the discrete time Markov chain converges weakly to a continuous-time Markov process described by:
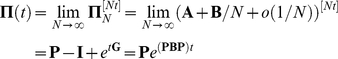
in which 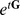 represents the matrix exponential and ***G*** is the infinitesimal generator or the rate matrix and is equal to the product of matrices ***PBP*** *=* ***G***.

We let 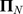 be the 26×26 transition matrix for the Markov chain describing the movement of two labeled lineages with the finite state space Ω. The entries of this matrix correspond to single time step transition probabilities between the elements of the state space. For example 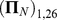 is the probability of coalescing in one time step given that the first and the second lineage are in the first age class. The full transition matrix 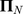 can be decomposed into two matrices ***A***, which represents the fast-time–scale events and ***B***, which represents slow-time–scale events. In biological terms, this decomposition is based on the fact that there are two fundamental types of events. The first type is the movement of lineages between age-classes. These happen with probabilities of *O(1)* per unit time. The second type is the coalescence event. Each time both lineages are in age class one they have a chance to coalesce with probability of *O(1/N).* Because of the order-of-magnitude difference in these probabilities, the lineages are expected to spend most of their time moving around different age-classes before eventually coalescing. The entry corresponding to the coalescence probability when both lineages are in the first age class can be calculated as:
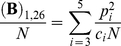



On the other hand the fast time-scale processes of movement between age classes will be determined by the matrix ***A*** which will have the following structure:
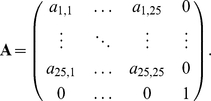
This matrix represents the transition matrix for a Markov chain for which the state space can be separated into two disjoint communicating classes. One of them includes the coalescence state and the other class includes all the other states. Consider the matrix 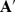 obtained by deleting the last row and column of the matrix ***A***. This matrix is ergodic since there is a non-zero probability of transition to all the other states in a finite number of time steps regardless of the initial state. Furthermore, if we let 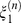 and 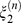 be the n-th power of the transition matrix describing the movement of a single lineage between age classes, then the n-step transition matrix 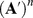 is equal to the Kronecker product of 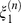 and 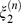. Then, we can write the following limit as the number of time steps tends to infinity:
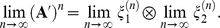



Recall that the Kronecker product between two matrices is a special case of tensor products. If *V* and *U* are two *m*-by-*n* matrices, the Kronecker product of these matrices denoted by 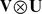 is equal to a *m* ^2^-by-*n* ^2^ matrix with the following block structure:
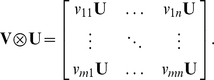
It is much easier to compute 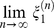 than to compute 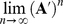 so using the Kronecker product simplifies the calculation of the matrix ***P***.

Since all of the entries of 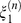 are positive recurrent for a finite *n*, there is a unique stationary distribution which can be obtained by considering the expected return time [41] and determined by the following vector:
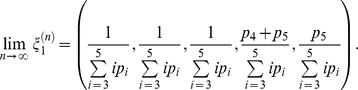
Given this stationary distribution, the existence of the limit matrix ***P*** follows directly. By obtaining the limiting matrix ***P***, for fast time-scale events, and the matrix ***B*** for the slow time-scale coalescence events, the weak convergence to Kingman's coalescent can be established using Möhle's convergence theorem.

We will not derive the matrix exponential explicitly, but instead, we will concentrate on obtaining the scaling factor that will reduce the system to the Kingman's coalescent. The inverse of this scaling factor times the population size *N* gives the coalescent effective population size. The coalescence rates correspond to the entries of the last column of the rate matrix ***G*** when time is measured in units of generation length. These entries can be calculated as
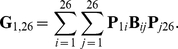
Since the 26^th^ column of matrix ***P*** is all zeros except for the entry in the last row, which is equal to one,
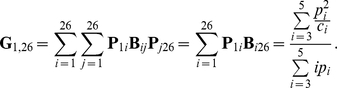



When time is scaled by the population size multiplied by the generation time, the coalescence times will follow an exponential distribution with rate given by this equation. Hence, one can rescale time by this factor and reduce this system to the Kingman's coalescent, in which the rate of coalescence is equal to one for a pair of lineages. When this rescaling factor exists, the coalescent effective population size is given by
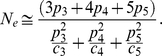
Thus, the effective population size is a function of the relative contributions of each age class and the relative mortality rate. Therefore, the age-dependent fecundity and expected generation time are the determinants of the effective population size in this population.

Some special cases are helpful in understanding the expression above. If all individuals live for exactly 5 years, the sole reproducing age class will be the fifth age class such that p_3_ = p_4_ = 0 and p_5_ = 1. The number of individuals in each age class will be equal and *N*_1_ = *N*_2_ = *N*_3_ = *N*_4_ = *N*_5_. Therefore, the proportion of individuals dying in the last age class will be equal to c_5_ = 0.2. The coalescent effective population sizes in this scenario and the standard Wright-Fisher model are equivalent and we have *N*_e_ = *N*.

In understanding the population dynamics of Pacific salmon, the CES has several advantages over the classical work on populations with overlapping generations. The classical approach is to approximate the variance in the contribution of a cohort to future generations and hence to calculate the variance effective size [42], [43]. Yet, in our particular system of interest, calculating the exact inbreeding coefficients require a greatly increased complexity [44] hence the practical importance of this approach is low in our model. Furthermore, the existence of the coalescent effective population size is a more general and stronger condition about the genetic structure as previously analyzed [16].

#### (iii) The Impact of Fast Fluctuations in the Population Size

Fluctuations in the population size from year to year are considered a major factor decreasing genetic variation and hence it is critical to address how these fluctuations could affect the long-term effective population size of salmon [30]. If the relative contribution of each year's breeders to the next generation is constant regardless of the fluctuations then the effective population size is roughly equal to the harmonic mean of the effective number of breeders (N_b_ = (c_3_+c_4_+c_5_)*N) per year [30], [37]. Yet, if the contributions to the next generation are directly proportional to the effective number of breeders then the multi-generation effective population size is approximated by their arithmetic mean [30]. Hence the demographic assumptions are strongly correlated with the effect of fluctuations in population size.

In this section, we relax the assumption of constant population size. Under a model of deterministically varying population size, where *g* is a function describing the population size fluctuations in time, the coalescence rates will be proportional to the inverse of the function *g*. Hence by accounting for this variation in the coalescence rates by a non-linear change in time-scale, it is possible to show convergence to the *n*-coalescent [45], [46], [47]. A more challenging problem is to allow stochastic variation in population size. It is helpful to classify these stochastic changes in the population size relative to the timescale that the coalescence events are taking place [16].

Stochastic fluctuations may occur on the same timescale as coalescence. In this case, the changes in the population size occur on an evolutionary timescale and when there is historical evidence for population expansion and/or shrinkage this type of modeling is appropriate. The variation in the population sizes is generally modeled by a discrete Markov chain, which itself can be approximated by a continuous-time process satisfying certain assumptions. Then the scaled ancestral process can be shown to converge to a stochastic time change of the Kingman's coalescent [48], [49].

Salmon populations fluctuate considerably from year to year, so we model the fluctuations in the population size as taking place on a fast time scale compared to coalescence, which takes place on the time scale of 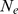 generations. We will denote the total population size by 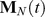 at year *t*. We assume that despite the fluctuations, the population size remains relatively large at all times such that 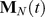 is proportional to a large population size *N* and 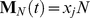 where *x*_j_ is a proportionality constant. The sequence of 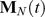
's for *t = (0, 1, …,)* represent the population sizes backwards in time with 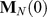 being the current population size from which two alleles are sampled. For simplicity, we also assume that the population size 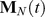 for all *t* to be independent and identically distributed random variables. We denote the probability that the population size at a given generation *t* to equal a specific value by ***q***_j_ such that
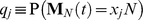
where *x*_j_ can take on finitely many values so that there are a fixed number of discrete and finite possible values for 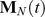. This assumption is possibly unrealistic since the population sizes at any given year probably depend on the sizes in the last few years. Yet, the methods we use in this section are general and with some effort can be applied to deduce the long term CES when the sequence of 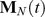 are modeled in a manner other than the simple case we consider.

The existence of the coalescent effective population size is quite powerful as one can directly apply previously developed tools of coalescent theory to the particular salmon model under consideration. This is a major advantage of using the coalescence effective size as opposed to inbreeding or variance effective population sizes since a separate analysis would be required to incorporate the fluctuations in the population size into these other measures of effective size. Our treatment follows Sjödin et al.'s (2005) discussion of the fast fluctuations in the standard Wright-Fisher model. An analysis of general reproduction models where the fluctuations in population size can be modeled by a first-order Markov chain can be found in [50].

To derive the long-term coalescent effective population size, the effect of fluctuations on the coalescence probability should be considered. All other demographic parameters of the original model are assumed to remain constant in time, and the relative sizes of the age classes grow and shrink in linear proportion to the total population size. In other words, *p_i_* and *c*_i_ are constant throughout the history of the sample and 
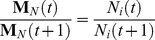
 for all *t* where 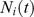 is the size of the i-th age class at time *t*. Then the probability of observing no coalescence events in 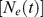 generations is given by
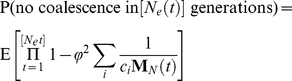
where φ is the proportion of the individuals in the first age class to the total population size. The independence of each year's population size and the results from previous section imply that
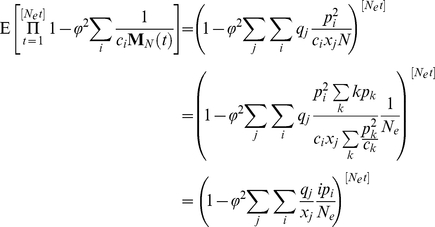
As we take the limit as 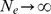,
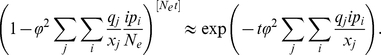
This result suggest that if time is rescaled by the coalescent effective population size as derived in the previous section the rate of coalescence will be equal to 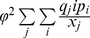. Recall that in the standard treatment of the Wright-Fisher model when time is rescaled by the population size, the coalescence rate is equal to one. To account for the discrepancy in the dynamics, the relevant multi-year coalescent effective population size 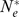 can be calculated as
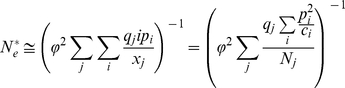
This result suggests that the long term coalescent effective population size is proportional to the harmonic mean of the population size at individual years, similar to the standard result for an unstructured Wright-Fisher population [51].

## Results

**Figure 2:**
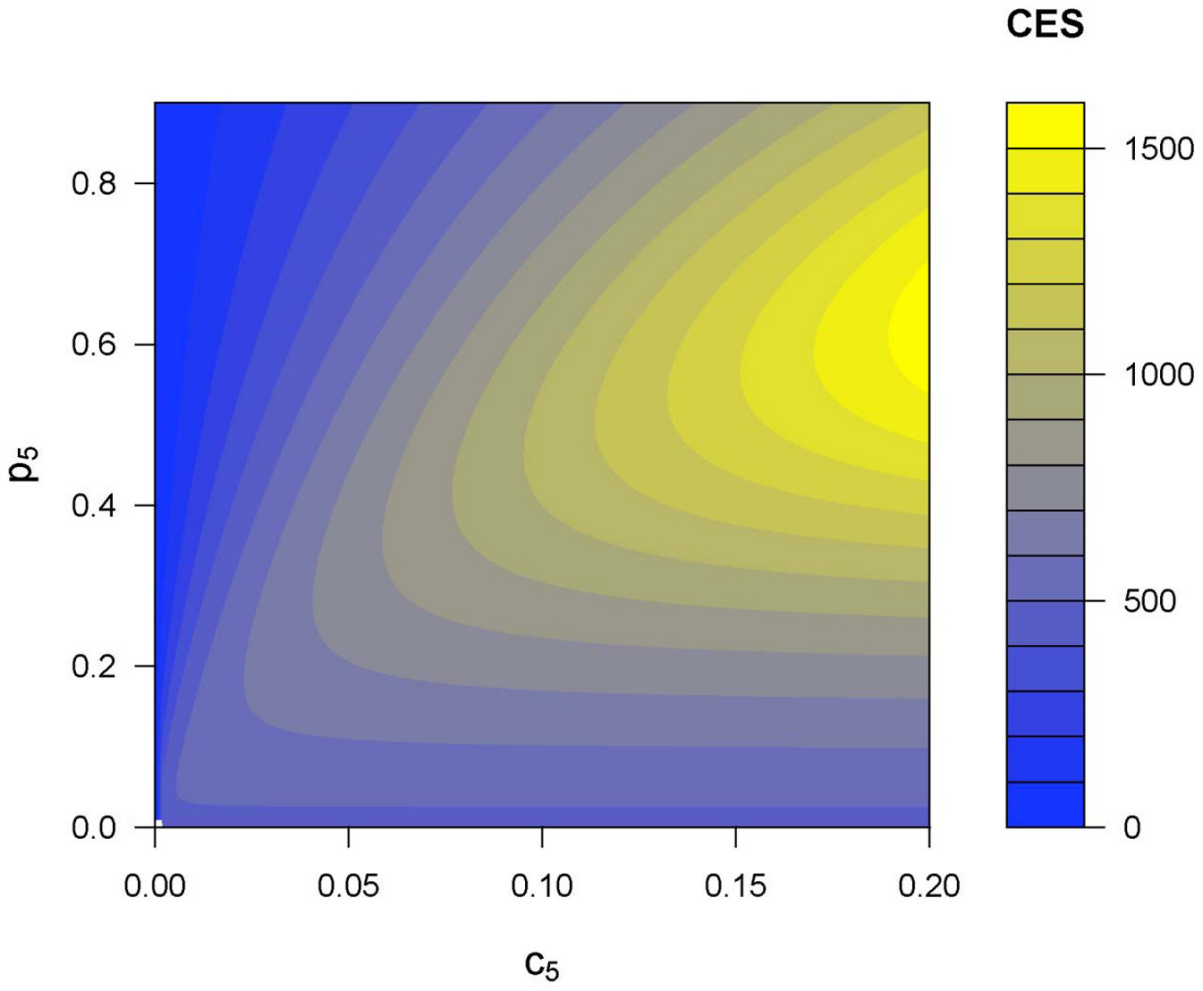
Coalescent effective population size as a function of the size of the last age class and the relative contributions to the first age class. The coalescent effective population size is graphed as *p_4_*, *p_5_* and *c_5_* are allowed to vary and the other parameters are fixed. The total population size is 1000 (*N* = 1000). The other parameters are c_3_ = 0.05, c_4_ = 0.1, p_3_ = 0.1 and p_4_+p_5_ = 0.9. The projection onto *p_5_*−*c_5_* plane was shown.

In natural populations of Chinook salmon, the contribution and the number of reproducing three-year-old individuals are generally smaller compared to the fourth and fifth age classes, and so these individual have comparably less influence on the overall genetic structure [34]. To explore the effect of variation in some of the parameters in the model, we fixed two parameters (*p_3_*, *c_3_*) and the relative number of individuals reproducing at age four (*c_4_*) at biologically relevant, representative values and analyzed the change in coalescent effective population size as we vary the other parameters (Figure 2). For a given value of age specific fecundity, increasing the relative abundance of reproducing individuals (*c_5_*) increased the CES. The increase in *c_5_* corresponds to an increase in generation length. The increase in CES as the generation length gets longer agrees with previous results using a forward-time genetic model [37]. On the other hand, for a fixed value of *c_5_* there is not a monotonically increasing relationship between CES and *p_5_*. A disproportionately large *p_5_* compared to *p_4_* would mean higher variance in reproductive success among age classes, as an individual in the fifth age class would contribute a disproportionately large number of offspring compared to an individual in the fourth age class. This is also in agreement with previous studies on sockeye salmon (*Oncorhyncus nerka*) and steelhead trout (*Oncorhyncus mykiss*) [52], [53], [54]. Similarly, these experimental studies suggested that variability in reproductive success is the main cause of low effective population size in these organisms using inbreeding effective population size.

We compared two previously known equations for estimating effective population size in populations with fluctuating size with the estimates from CES derived in the previous section. The first equation is derived under the assumption that spawners in each year contribute equally to the next generation regardless of their abundance and is simply given by 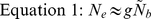
where *g* is the generation length and 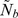 is the harmonic mean of effective number of breeders in individual years [30], [37]. The second equation can be derived under the assumption that each year's spawning population contributes to the next generation in one to one proportion to the number of spawners and is given by
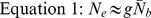
where 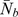 represents the arithmetic mean of the effective number of breeders in the individual years [30], [37].

**Figure 3:**
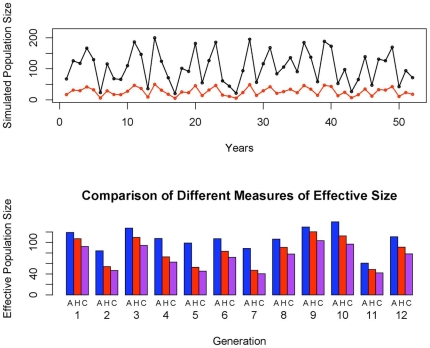
Comparison of different estimates of effective population size in response to fluctuation in population size. (A) A time-series of simulated effective population sizes for 52 years is shown. The simulations were done by uniformly sampling from the interval between 20,000 and 200,000. Each unit on the y-axis represents 1000 animals. The black line corresponds to the simulated population size *N*, the red line is the approximate number of effective number of breeders N_b_ = (c_3_+c_4_+c_5_)*N. The arithmetic mean and harmonic mean are calculated using the effective number of breeders. (B) The simulated population sizes are divided into 12 generations, each with a 4 year length. For each generation the effective population size is calculated using three different methods. A: Arithmetic Mean (Equation 2), H: Harmonic Mean (Equation 1) C: Coalescent Effective Population. Each unit on the y-axis represents 1000 animals as in panel A.

We compared the predictions of our estimate of effective population size with the predictions of Equations 1 and 2. We simulated a time-series of population sizes for 52 years. For spring chinook salmon, the total number of returns to the Columbia River system in the last fifty years ranged between 20,000 to 200,000 individuals [34]. We used these figures as estimates of the upper and lower bounds for the total population size *N*. Specifically, we generated the population sizes by sampling uniformly from the interval between 20.000 and 200.000. In the calculation of the long term CES, we used the following parameters: *p*_3_ = 0.04, *p*_4_ = 0.25, *p*_5_ = 0.71, which correspond roughly to the mean frequencies of spawners of ages 3, 4, and 5 respectively in the Marsh Creek population of spring chinook salmon years [30], [34]. By choosing this set of parameters, we made the assumption that the relative contribution of the spawners in each age classes to the first age class is equal to the mean frequency of the breeding individuals in each age class. We chose φ = 0.5, *c*_3_ = 0.01, *c*_4_ = 0.05, *c*_5_ = 0.15 which are reasonable estimates for the salmon life history and also results in roughly equivalent variance across age classes in the number of offspring per breeding individual. The results of the comparisons between the different measures of effective size for a particular set of simulated population sizes are given in Figure 3. Under a wide range of parameters that are plausible for salmon, the qualitative behavior of the different estimates of the coalescent effective size was essentially the same as long as φ was greater than or equal to 0.5 (data not shown). Previously, Waples' computer simulations suggested that effective population size agreed better with the harmonic mean method and was generally lower than both the harmonic and the arithmetic mean methods [30] and our results agree with this observation (Figure 3).

## Discussion

We have shown that for certain models of semelparous age-structured organisms, the ancestral process for a sample of size two converges to a continuous-time process as in Kingman's coalescent, if time is rescaled appropriately. We have established this result using a separation of time scales approach combined with a novel method using Kronecker products to simplify the calculation of the limit process. This convergence holds in the limit as the total population size tends to infinity and the sizes of the age-classes relative to the total population size remain constant. The use of the Kronecker product enables direct generalization of our results for a sample size of *n*, so long as sample size is small such that multiple coalescence events are negligible. The latter point is essential to proving that the coalescent effective size exists [55]. Although we state it without proof, in our model, multiple coalescence events will not occur in the limit because we assume Wright-Fisher reproduction and the number of potential parents is proportional to *N*.

For concreteness, we analyze a model with five age classes out of which two represent juvenile age classes. However, the theory can be generalized in a straightforward manner to a general finite number *k* of age classes, some of which are non-reproducing. A *k* by *k* transition matrix ξ for the ancestral process of a single lineage should be considered and the Kronecker product of two of these transition matrices needs to be taken to obtain the fast-process matrix in the decomposition of the Markov chain for the ancestral process of two lineages. The calculation of the effective population size will then be essentially the same as in our model.

Coalescent theory has recently been extended to incorporate the consequences of evolutionary factors such as recombination [56], [57], selection [58], [59] and spatial subdivision of populations [60], [61], [62], [63], [64] among other things. Our study has shown that for certain models of semelparous age-structured organisms, the ancestral process for a sample of size two converges to Kingman's coalescent with appropriate rescaling of time. Consequently, our result enables many of these previously developed tools of coalescent theory to be applied to address questions regarding salmon population biology. As an illustrative example we demonstrated how the rapid fluctuations in population size would affect the long-term behavior of the population.

For mathematical tractability, we have assumed that the age structure in the population is stable and the relative contribution of the age classes to the newborns is constant over time, even as the size of the population changes. We also assumed that within each age class the reproducing individuals have a symmetric joint multinomial distribution of their offspring. In natural populations the variance in the number of offspring could be very high and this would result in a very different ancestral process [65], [66], [67], [68], [69]. In addition, the relative sizes of the different age classes might change over time. Therefore, it is of interest to test the validity of these assumptions about age structure and reproductive variance. If these assumptions do not hold, one might expect a smaller effective population size compared to the harmonic mean since the time to the common ancestor would be shorter.

The model we analyzed has several other simplifying assumptions. We assumed a haploid population structure but our results should hold for a diploid population as long as there are no major differences in age structure or variability in reproductive success among the sexes [22]. Yet, these are known to be variable among sexes in natural populations; for example the spawners in age class three were predominantly males in spring chinook salmon population from Marsh Creek [34]. An extension of the coalescence model we analyzed here could be used to address the possible effects of such sex-specific differences.

In our model, we also assumed no subdivision of the population. In fact, the estimates of population sizes we used in our simulations correspond to the metapopulation size of chinook salmon that are destined to spawn in numerous local populations. Even though we don't have a clear understanding of the extent of genetic material exchange between these subdivided populations, a detailed analysis of the potential effects of population subdivision would be essential. Coalescent theory has been successfully applied to study the effects of spatial subdivision of populations [59], [60], [61], [62], [63] and our work should enable future studies to address these issues for the case of Pacific salmon.

Our analysis of semelparous age structure also reveals a connection between this model and the strong migration limit which is concerned with migration among demes [23], [70]. In the strong migration limit, the population is divided into a finite number of colonies or demes and the size of each deme is assumed to be large. The movement between demes is described by a backwards migration matrix such that the *ij*-th entry of the matrix corresponds to the probability that an individual in deme *i* migrated from deme *j* where *i* and *j* are not necessarily distinct. Strong migration assumption implies that random drift is much weaker than migration when the population size tends to infinity. In both our model and the strong migration limit, coalescence takes place on much more slower time scale compared to the movement between age classes and migration respectively.

Under strong migration, the effective size is equal to total population size when the migration is conservative, which means that at each time step the number of individuals leaving any given colony and the number of individuals arriving to the same colony is equal [70]. In our model, an analogous result holds for multiple parameter sets but one particular choice of parameters that results in *N*_3_ = *N* is *p*_3_ = 3c_3_, *p*_4_ = 4c_4_, *p*_5_ = 5c_5_. This particular set of parameters implies that if the relative contribution of the age classes is weighted appropriately to the relative numbers of reproducing individuals then the coalescent effective size is equal to the population size. This conclusion is in agreement with previous analysis using forward-time population genetic models [30].

Our model for Pacific salmon captures the life history traits similar to those seen in plants with seed banks. Various models that try to capture the dynamics of these seemingly unrelated but fundamentally similar organisms have been previously compared [39]. Kaj et al. (2001) [71] developed a coalescent model for plants with seed banks and found that the scaled ancestral process in that model also converges weakly to the Kingman's coalescent with time a change under the assumption of constant population size. The ancestral process for seed banks also includes both a fast time-scale “configuration process” and coalescence events in a slow time scale. In contrast, our model is specifically tailored to the dynamics of Pacific salmon populations, allowing for different sizes in different age classes as well as the more realistic assumptions of presence of juvenile age classes, and allowing only a subset of individuals in a given age class to reproduce. We also obtained a simple expression of the coalescent effective population size and extended our results to the case of variable population size.

Kobayashi and Yamamura (2007) [72] have considered an age-structured population where the movement between age classes is similar to that in our model. They analyze spatial structure where each deme has age structure. They derived an effective population size although their system does not converge to Kingman's coalescent. The main difference between their study and ours is the assumptions regarding the reproductive scheme. They have assumed that reproduction is similar to the standard age-structured model [73], [74].

In this work, we have derived a simple expression for the coalescent effective size for a different reproductive scheme that captures the life cycles of semelparous organisms. The existence of the coalescent effective size readily allows extensions to include the effects of various evolutionary forces such as rapid fluctuations in population size. Our results could also be extended to explore the possible effects of spatial structure on Pacific salmon populations.
